# Coherent control of a strongly driven silicon vacancy optical transition in diamond

**DOI:** 10.1038/ncomms14451

**Published:** 2017-02-20

**Authors:** Yu Zhou, Abdullah Rasmita, Ke Li, Qihua Xiong, Igor Aharonovich, Wei-bo Gao

**Affiliations:** 1 Division of Physics and Applied Physics, School of Physical and Mathematical Sciences, Nanyang Technological University, 21 Nanyang Link, Singapore 637371, Singapore; 2 School of Mathematical and Physical Sciences, University of Technology Sydney, Ultimo, New South Wales 2007, Australia; 3 Faculty of Science, Institute of Biomedical Materials and Devices (IBMD), University of Technology Sydney, Ultimo, New South Wales 2007, Australia

## Abstract

The ability to prepare, optically read out and coherently control single quantum states is a key requirement for quantum information processing. Optically active solid-state emitters have emerged as promising candidates with their prospects for on-chip integration as quantum nodes and sources of coherent photons connecting these nodes. Under a strongly driving resonant laser field, such quantum emitters can exhibit quantum behaviour such as Autler–Townes splitting and the Mollow triplet spectrum. Here we demonstrate coherent control of a strongly driven optical transition in silicon vacancy centre in diamond. Rapid optical detection of photons enabled the observation of time-resolved coherent Rabi oscillations and the Mollow triplet spectrum. Detection with a probing transition further confirmed Autler–Townes splitting generated by a strong laser field. The coherence time of the emitted photons is comparable to its lifetime and robust under a very strong driving field, which is promising for the generation of indistinguishable photons.

## Abstract (web summary)

Silicon vacancy centres in diamond have been identified as potential highly efficient solid-state qubits for on-chip integration. Here, Zhou *et al*. demonstrate coherent control of silicon vacancy centres in nanodiamonds and observe Autler-Townes splitting, Mollow triplet and Rabi oscillations.

Coherent control of atom–photon interfaces is vital in the realization of quantum information protocols. In particular, the interface between solid-state qubits and photons is a promising candidate for practical and scalable quantum technologies^1^,^2^,^3^,^4^,^5^. Different types of defects in solids have been studied so far and many of them show excellent optical or spin properties to be proper quantum qubit candidates. Among others, the nitrogen vacancy (NV) centres in diamond have achieved partial success in spin–photon interface and spin–spin entanglement mediated by photons^1^,^2^. However, despite the long spin coherence times of the NV, the defect suffers from low percentage (3–5%) of the total emission into its weak zero phonon line (ZPL) and from strong inhomogeneous broadening. Recently, increased efforts have been made to identify other solid-state qubits with improved inherent properties. The silicon vacancy (SiV) centre in diamond is one potential candidate, with ∼70% of the photons emitted into the ZPL^6^,^7^. Different SiV defects exhibit intrinsically identical spectral properties in low-strain bulk diamond^8^,^9^ and nearly transform-limited linewidth^9^,^10^,^11^,^12^. SiV spin coherence times are in the range of ∼40 ns at cryogenic (4 K) temperatures^13^,^14^. It is important to note that the coherence times are only limited by spin relaxation time and are estimated to increase dramatically at lower temperatures^15^. As a result, the SiV defect has become a promising candidate to be a key building block for quantum information processing.

Towards this goal, preparation and coherent control of the emitted photons from SiV is a prerequisite. For a two-level system under strong pumping resonant field excitation, state population will oscillate between the ground and the excited states, also known as Rabi oscillations. In the frequency domain, the emission under continuous wave (cw) laser excitation will exhibit the Mollow triplet spectrum^16^, which is a hallmark for quantum coherent control and enables a robust approach to generate single photons with detuned frequency from the resonance^17^,^18^,^19^,^20^,^21^,^22^,^23^,^24^,^25^.

In this work, we demonstrate coherent control of a strongly driven single SiV defect in diamond. In particular, we observe Rabi oscillations with fast photon detection using both a nanosecond laser pulse and a cw laser field. A Fourier transform of the time-resolved detection combined with laser detuning results in an observation of the Mollow triplet spectrum. In the frequency domain, the Autler splitting has been confirmed by a Λ-shape energy scheme^13^,^14^. In the above measurements, photon coherence, which is critical for photon interference in building a quantum network, has been characterized in both the low- and the high-power regime.

## Results

### Transitions in a single SiV in diamond

**Figure 1:**
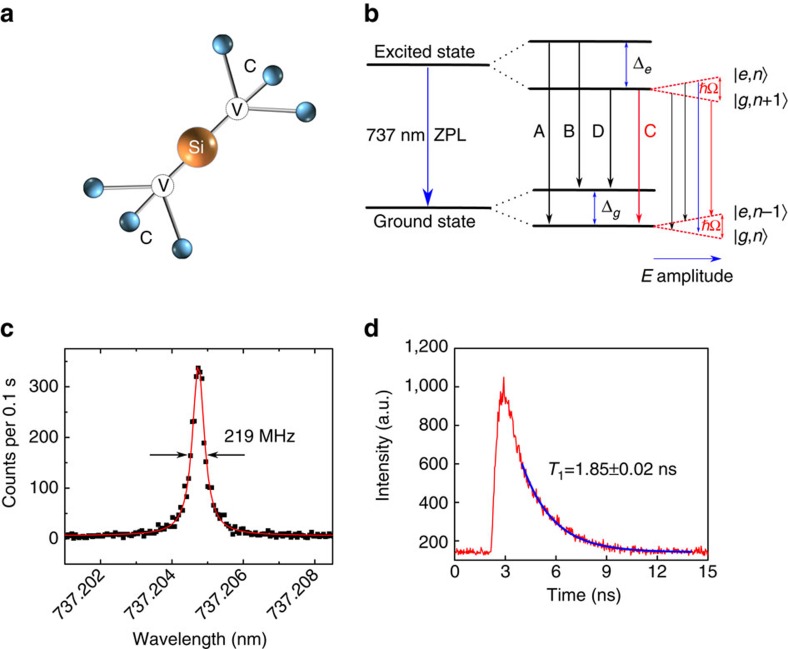
The SiV defect. (**a**) Atomic structure of a SiV colour centre in diamond. A silicon atom (yellow) is neighbouring two carbon vacancies (labelled V) in a diamond lattice. (**b**) Mollow triplet and SiV energy level scheme. The ZPL is at 737 nm. The excited and ground states split by an energy Δ_*e*_=410 GHz and Δ_*g*_=228 GHz, respectively, resulting in four transitions labelled as *A*, *B*, *C* and *D*. With increasing electric field (red) coupling to *C* transition with a resonant laser, four dressed states are formed and labelled as |*g*,*n*〉, |*e*,*n*−1〉, |*g*,*n*+1〉 and |*e*,*n*〉. As the *E* amplitude increases, the splitting of the dressed states (*ħ*Ω) also becomes larger. (**c**) Resonant measurement of the *C* transition linewidth at saturation power, the raw data (black) are fitted by Lorentzian function (red). (**d**) Lifetime measurement. A resonant laser pulse of ∼100 ps is used to excite the *C* transition, resulting in a decay (red) of the emitted photons in the time domain, a lifetime of 1.85 ns is extracted from an exponential fitting (blue).

The SiV defect consists of an interstitial silicon atom, neighbouring two vacancies along a [111] crystallographic axis in diamond^26^, as shown in Fig. 1a. The defect is negatively charged and has a *D*_3*d*_ symmetry, which is inert to strain and fluctuating electric fields^27^,^28^,^29^. The electronic structure and optical transitions of the negatively charged SiV in diamond have been characterized in detail recently^28^,^29^,^30^. Both ground and excited states of the SiV ZPL are split due to spin–orbit coupling, resulting in four lines at cryogenic temperature as shown in Fig. 1b (black solid lines). In our experiment, the transitions are successfully identified by scanning a resonant laser and recording the photoluminescence at the same time (see Supplementary Fig. 1). For the current work, coherent control is performed on transition *C* as marked in Fig. 1b. Transition *C* is associated with the lower energy levels in both the excited state and ground state in SiV. It does not suffer from fast relaxation from the upper branches of the energy levels, therefore offering a better count rate and longer coherence times compared with the *A*, *B* and *D* transitions. By resonantly exciting transition *C* and collecting the photons at the phonon sideband above 750 nm, a narrow linewidth of 219 MHz at saturation power is achieved (Fig. 1c). The linewidth without power broadening is 154 MHz, close to the transform-limited linewidth of 86 MHz, calculated from a direct lifetime measurement that is shown in Fig. 1d.

### Time-resolved Rabi oscillations

We first demonstrate real-time observation of the time-resolved Rabi oscillations measured with a superconducting detector with a short jitter time. In the time domain, a strong resonant cw laser coupled to a two-level system yields oscillations of the excited state population and can be described by sin^2^(Ω_*g*_*t*/2), where 
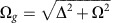
, where Δ is the laser detuning, Ω=*μE*/ℏ is the bare Rabi frequency, *μ* is the optical transition dipole moment and *E* is the driving field amplitude. In the frequency domain, the physics model can be best demonstrated with a dressed state as shown in Fig. 1b (red dashed lines). Under a strong laser field, the ground state and excited state will split (Autler–Townes splitting) into two states separated by an energy 
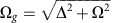
. Two frequencies of the emission from four transitions are degenerate and therefore the dressed state will result in an emission pattern with three Lorentzian lines with centre frequency *v*_0_+Δ and *v*_0_+Δ±Ω_*g*_.

**Figure 2:**
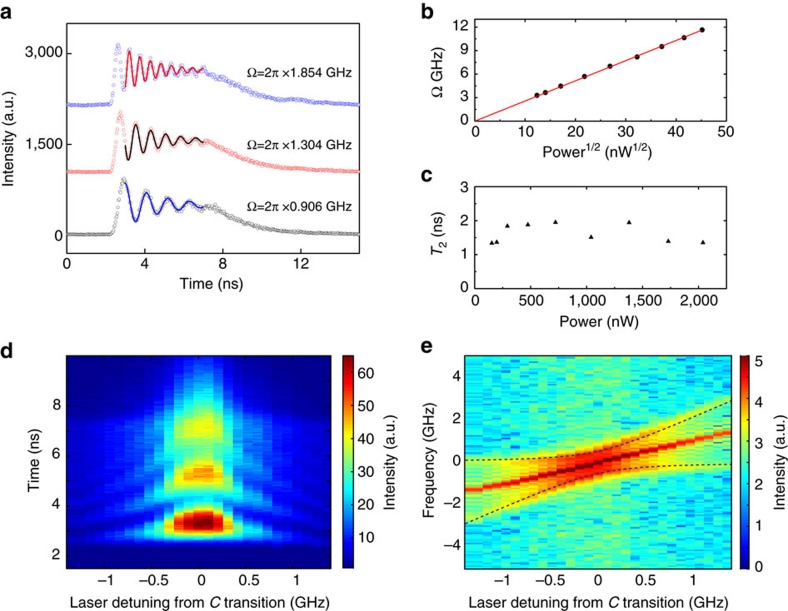
Time-resolved Rabi oscillations. (**a**) Rabi oscillations of the *C* transition when excited with a 5 ns laser pulse at three different excitation powers. Laser pulse period is 15 ns. Fitting of the curves with equation (1) results in the Rabi frequency Ω/2*π*=0.906, 1.304 and 1.854 GHz. (**b**) Fitted Rabi frequency for curves in **a** as a function of the square root of the excitation laser power, exhibiting a linear trend (red line). (**c**) The extracted *T*_2_ times from the fitting as a function of the excitation laser power. No apparent decrease is seen up to 2,000 nW, which corresponds to 300 times the saturation power. (**d**) Excitation with a fixed Rabi frequency Ω=2*π* × 1.304 GHz, while modifying the laser detuning from *C* transition. Different colours represent different intensity amplitude. (**e**) Fourier Transform analysis of the measurement in **d**. Two side branches fit well with the theoretical values 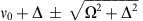 (dashed black lines).

In our experiment, a 5 ns laser pulse is used to excite a single SiV centre. The emitted photons are collected and analysed with a time-correlated single-photon counting system (see Methods). As shown in Fig. 2a, the Rabi oscillations are clearly observed with a shorter oscillation period corresponding to a higher excitation power. The curves are fitted with the theoretical value of excited state population, which can be expressed as (up to a displacement time *dt*)^19^


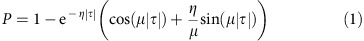


Here *η*=1/2*T*_1_+1/2*T*_2_ and 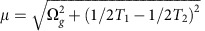, where *T*_1_ and *T*_2_ are the lifetime and the photon coherence time, respectively. In the fitting, *T*_2_ and Ω_*g*_ are used as free parameters, while the measured *T*_1_ value is 1.85±0.02 ns. The extracted Rabi frequency is linearly proportional to the square root of the excitation power, confirming the Rabi oscillation behaviour (Fig. 2b). Average value of *T*_2_ is calculated to be 1.62 ns, smaller than the ideal value 2*T*_1_=3.7 ns. This is likely to be due to the pure dephasing of the excited state. More importantly, as shown in Fig. 2c, the value of *T*_2_ stays above 1.3 ns even with a Rabi frequency of 12 GHz, corresponding to 300 times of saturation power. This shows that no apparent excitation induced dephasing is observed. This can be partially explained by the weak electron–phonon coupling of the SiV and the reduced sensitivity to fluctuating electric fields within the diamond lattice^8^,^9^,^10^,^11^,^12^. Robust *T*_2_, combined with the frequency stability and efficient generation of SiV single photons, shows the feasibility of SiV as an efficient and coherent single photon source.

The plot of the time-dependent fluorescence intensity profile as a function of laser detuning is shown in Fig. 2d. With the laser detuning, the oscillation frequency is increased to the generalized Rabi frequency 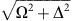. To best illustrate this phenomenon, a Fourier transform of each curve is taken and the frequency components are shown as a function of the laser detuning. Three peaks corresponding to the three frequency components of the Mollow triplet can clearly be seen. The dashed lines in Fig. 2e corresponds to the theoretical centre frequency 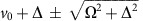 with Ω extracted when the laser detuning is 0.

### Photon correlation measurement

**Figure 3:**
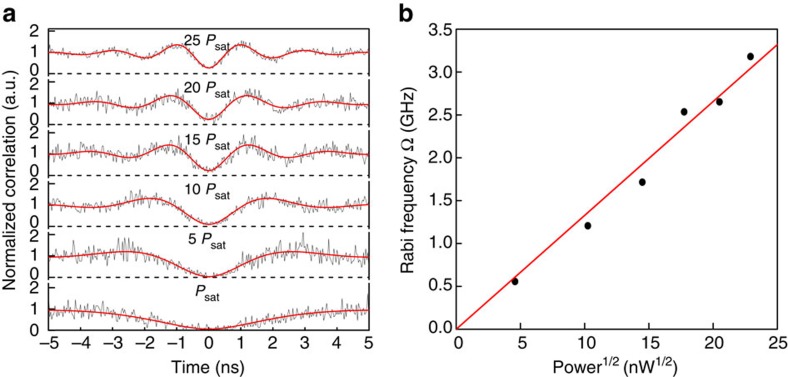
Photon correlation measurements. (**a**) Raw data (black)of the second-order correlation function *g*^(2)^(*τ*), fit with equation (1) (red). The *g*^(2)^(*τ*) is normalized to 1 at infinite delay times. *P*_sat_ is the saturation power ∼20 nW. *T*_1_=1.85±0.02 ns and *T*_2_=1.62 ns are used, while Ω is fitted. (**b**) The Rabi frequency as extracted from the fitting is plotted as a function of the square root of the excitation laser power. The values follow a linear trend, indicated by the red line.

To study the quantum nature of a single SiV defect in more detail, the second-order correlation function *g*^(2)^(*τ*)=〈*I*(*t*)*I*(*t*+*τ*)〉/〈*I*(*t*)〉^2^ is recorded as a function of excitation power using a Hanbury–Brown–Twiss setup. For a perfect single-photon emitter, *g*^(2)^(0)=0. In our experiment (Fig. 3a), the resonantly excited SiV shows a *g*^(2)^(0)=0.061±0.026 at saturation power, without correcting for the detector response time. When the transition is driven strongly (above saturation), oscillations with different periods are observed in the delay time range of [0, ±5] ns. Unlike the single-photon detection experiment in the above paragraphs where the time 0 is set by the excitation pulse rising time, here the start signal is set by the detection of the first emitted photon in the *g*^(2)^(*τ*) measurement. The first photon (start signal) prepares the system into the ground state and the second photon (stop signal) will show the population of the excited state, which varies due to the Rabi oscillations. The measured *g*^(2)^(*τ*) data (Fig. 3a) are fitted with the same equation (1) up to a scaling factor, *A*, and displacement count Δ*g* due to the effect of background and the impulse response function of the finite detector. A single set of *T*_1_=1.85±0.02 ns and *T*_2_=1.62 ns fits the data well at different excitation powers. The Rabi frequencies extracted from the fitting are plotted as a function of the square root of the laser power, showing an expected linear relationship (Fig. 3b) comparable to the one obtained from the time-resolved Rabi oscillations experiment.

### Autler–Townes splitting

**Figure 4:**
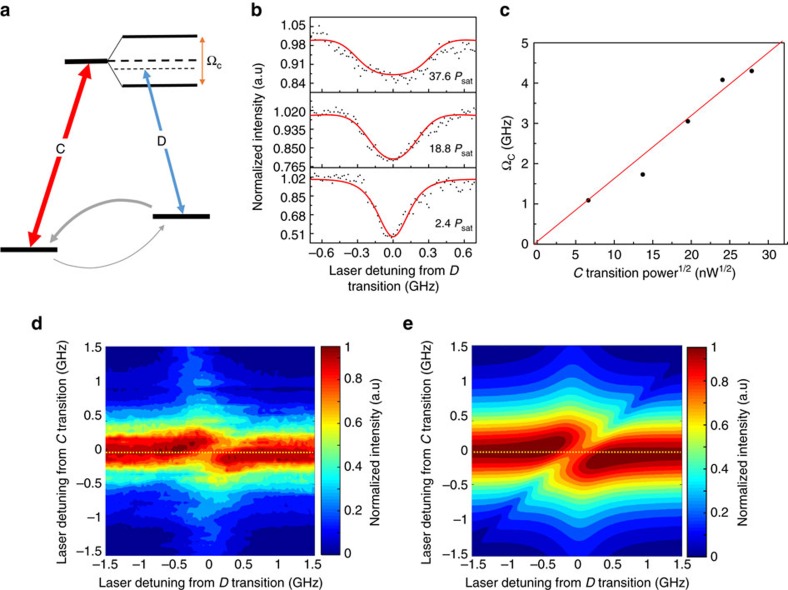
Autler–Townes splitting detection. (**a**) Level structure of the Autler–Townes splitting detection scheme. A much stronger laser is resonantly pumping the *C* transition while a probing laser is detuned on the *D* transition to detect the excited energy levels. Grey solid arrows represent the decay or relaxation from each level. (**b**) Experimental (black) and simulated curves (red) for three different laser powers exciting the *C* transition. The frequency of the excitation laser is fixed, and a second laser scans the *D* transition in the range of [−0.7 GHz, 0.7 GHz] from resonance. (**c**) Ω_*C*_ is extracted from the fit, and plotted as a function of the square root of power of the excitation laser (black dot), the red line is a linear fitting of the extracted data. (**d**) Experimental data for measuring the Autler–Townes splitting. SiV fluorescence counts are detected as a function of scanning the excitation laser frequency across the *D* transition in the range of [−1.5 GHz, 1.5 GHz] from resonance, whereas the excitation laser on C is also fixed and stepped in the range of [−1.5 GHz, 1.5 GHz] from resonance. Laser powers are 20*P*_sat_ and 2.5*P*_sat_ for *C* and *D* transition respectively, the yellow dashed line indicates the same condition as **c** when the *C* transition is exactly on resonance. (**e**) Simulated SiV fluorescence for the experiment in **d** (for simulation method and values, see Supplementary Note 1).

In addition to the Fourier transform analysis of the time-resolved Rabi oscillations, the Autler–Townes splitting can be observed in the frequency domain. To demonstrate this, the same transition *C* is resonantly strongly driven and is probed using transition *D*. The energy level structure to probe the splitting is shown in Fig. 4a. Consider a Λ system that includes a strongly pumped transition C (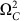 much larger than *P*_sat_) and a weakly probed transition *D*. The *C* and *D* transitions have different ground states and share the same excited state. A pump laser is tuned to the *C* transition and a probe laser is scanned across the *D* transition. The detuning of the laser relative to the *C* (*D*) transition is indicated by Δ_*C*_(Δ_*D*_)and the Rabi frequencies of the *C* (*D*) transitions are denoted as Ω_*C*_(Ω_*D*_). Similar to the coherent population trapping, the fluorescence intensity will show a dip around Δ_*D*_=0 due to the destructive interference between the dipole moment of transition *C* and *D*. If one of the transitions is strongly pumped, the splitting related to the observed dip will correspond to the splitting of the excited state also known as Autler–Townes splitting.

In the experiment, we vary the laser power for the *C* transition, whereas the power of the probe laser for the *D* transition is fixed. The normalized fluorescence intensity as a function of Δ_*D*_ is shown in Fig. 4b. The value of Ω_*C*_ is obtained by fitting the data with the Lindblad master equation (details can be found in Supplementary Note 1, Supplementary Fig. 2 and Supplementary Table 1). The value of the dip width is larger, as the power pumping on transition *C* increases, consistent with the Autler–Townes splitting. The Ω_*C*_ values extracted from the fitting are plotted as a function of the square root of the laser power, showing an expected linear relationship (Fig. 4c) comparable to the one obtained from the time-resolved Rabi oscillation experiment. To get more information on the fluorescence characteristics as a function of the laser detuning, intensity measurements are recorded by varying Δ_*C*_ and Δ_*D*_, while keeping the *C* transition excitation power fixed at ∼20*P*_sat_. The measured data are shown in Fig. 4d, where the yellow dotted line is a guide to the eye and the data are in good agreement with the simulated values (Fig. 4e).

### Rabi oscillations and Ramsey fringes with pulsed excitation

**Figure 5:**
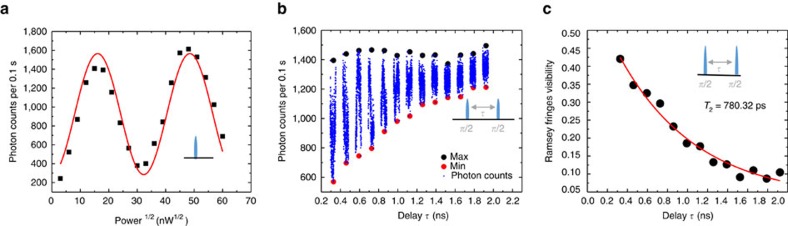
Ultrafast control of the SiV optical transition. (**a**) Rabi oscillation with pulsed excitation. The photon counts (black) are recorded as a function of square root of power, the raw data are fitted using a sine function (red). (**b**) The photon count oscillates when the delay between two *π*/2 ps pulses varies by *τ*. Maximum and minimum value of the Ramsey oscillations can be extracted as shown by black and red dots. Blue curves represent the oscillation of the photon counts around delay *τ* with time shown stretched here for illustration. (**c**) Ramsey fringe visibility as a function of delay *τ*. An exponential decay fitting (red curve) yields *T*_2_=780±141 ps.

In the last part, we demonstrate coherent control of SiV optical transition with a pulsed laser. To avoid cross-excitation of other unwanted transitions, a 200 ps laser with 80 MHz repetition rate is generated after a two-stage filtering setup based on a spectrometer and a fused silica etalon (Light machinery, FSR 30 GHz, Finesse 20, see Methods for detailed setup). As shown in Fig. 5a, the photon counts show Rabi oscillations with the increase of the laser power coupled to the optical transition. As expected, the oscillation follows a sine curve as a function of the square root of the excitation power. Furthermore, two *π*/2 pulses with a delay *τ* are used to measure the Ramsey fringes. The photon counts oscillate as the delay between the two *π*/2 pulses changes. As shown in Fig. 5b, we extract the maximum and minimum of the Ramsey fringe envelopes around delay *τ*. The visibility of the oscillation as a function of the delay *τ* is shown in Fig. 5c. With *τ* larger, the visibility will be smaller, which is related to both fluorescence lifetime and dephasing rate 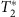. An exponential decay fitting of the visibility of the Ramsey fringes yields *T*_2_=780±141 ps, which is consistent with the linewidth measurement for this transition.

## Discussion

We demonstrated coherent control of a strongly driven optical transition of a single SiV defect in diamond. The SiV defects studied in this work are embedded in nanodiamond particles. Unlike the NV centre that only exhibits excellent quantum properties when embedded in a large ultra-pure bulk crystal, the availability of suitable quantum systems in nanodiamonds offers a great advantage. For instance, it opens up possibilities for engineering scalable hybrid quantum photonic systems by manipulating the nanodiamond hosting the SiV defect onto a photonic crystal cavity or a waveguide to engineer quantum registers. The coherence time of SiV emission is long and robust even at high excitation powers. This aspect is important for connecting quantum nodes through Hong–Ou–Mandel interference experiments^31^. Several immediate research directions emerge in the next steps: the Mollow Triplet photons in each sideband can be separated by the combination of a Michelson interferometer and Fabry–Perot filters^32^. The role of the fabry-perot filter (FB) is to filter out the excitation laser and produce a frequency tunable single-photon source by detuning the excitation laser frequency. A detailed study of the photon statistical signature can be made for the triplet emission^32^. In addition, the coupling of a Mollow triplet sideband to an optical cavity may lead to a strongly enhanced resonant sideband^33^,^34^,^35^, enabling a tunable dressed state laser^36^. Finally, under magnetic field the spin-resolved Mollow triplet may assist in single-shot spin readout^25^ or spin-photon interfaces.

## Methods

### Experimental setup

The sample was mounted on a 4 K cryostation (Montana Instruments). A home-built confocal microscope system was used to excite the sample and collect the fluorescence through a 0.8 NA Nikon objective. In the photoluminescence excitation experiment, a titanium:sapphire (Ti:sapph) laser with <5 MHz linewidth was used to excite the sample. The phonon sideband (>750 nm) of the emission was collected and guided to an avalanche photo diode through a single-mode fibre. In the photon correlation antibunching measurement, the emission was guided to a beam splitter. Two avalanche photo diodes were used to record the counts and connected to a time-correlated photon counting card (Picoharp, PH300). In the time-resolved Rabi oscillation experiment, an arbitrary waveform generator (Technotronix AWG7000) was used to generate the pulse triggering an electro-optic modulator and to synchronize the whole experiment. The emission was guided to a low jitter (∼30 ps) superconducting single-photon detectors. For the Autler–Townes splitting detection, two lasers (Ti:sapphire laser and a diode laser) were combined together using a beam splitter and then guided to the excitation arm. All lasers were locked to a wavemeter with 10 MHz accuracy and feedback speed of 500 Hz, to avoid centre wavelength drift. In the time-resolved Rabi oscillation, photon correlation and Autler–Townes experiment, we collected the emission with polarization perpendicular to the input laser, to suppress residual counts from the excitation laser.

### Two-stage filtering system

**Figure 6:**
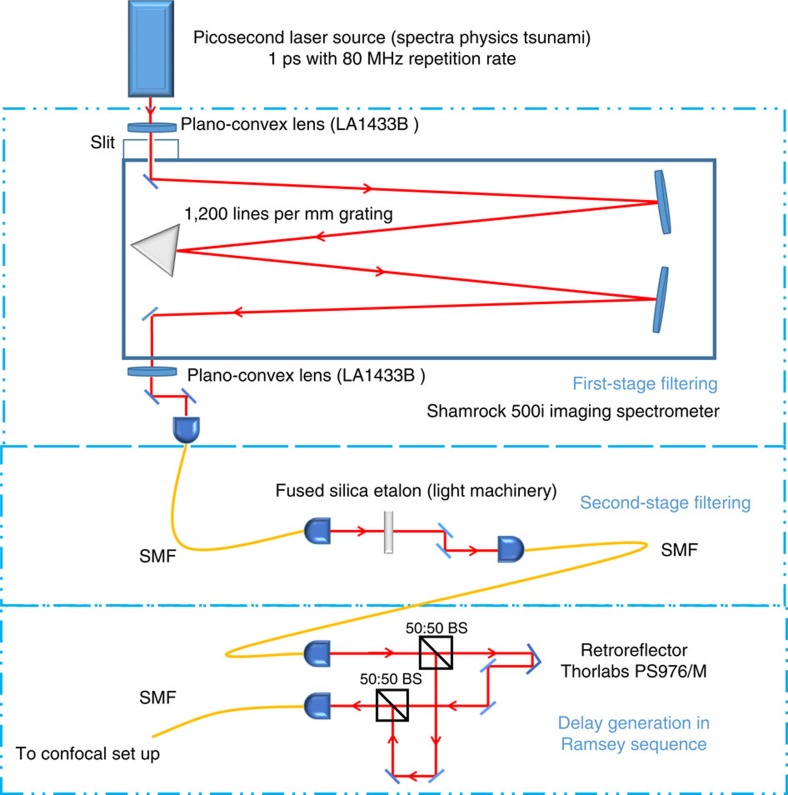
Schematic of the two-step filtering system. The picosecond laser was first filtered by an Andor Shamrock 500i spectrometer system with a 1,200-line-per-mm grating and further narrowed by a fused silica etalon. After two-stage filtering, the linewidth of the pulsed laser was around 1.65 GHz. The third part was used to generate the delay between two pulses in the Ramsey sequence. The beam was first split by a 50:50 beam splitter (BS). One arm of the beam was delayed and combined with the other undelayed pulse by another 50:50 beam splitter. The beam was guided by single mode fiber (SMF).

The two-stage filtering system is used to produce the 200 ps pulse lasers from a 1 ps Ti:sapphire laser. The narrowing of the laser transition helps to avoid multi-excitation of other unwanted SiV transitions. The two-stage filtering system used in the picosecond-excitation experiment is shown in Fig. 6. In the first stage, the picosecond laser was filtered by an Andor Shamrock 500i spectrometer system. After the first-stage filtering, the picosecond laser was narrowed to 16 GHz. The second-stage filtering was based on a fused silica etalon (FSR 30 GHz, Finesse∼20) and after filtering, the picosecond laser was narrowed to 1.65 GHz, which is sufficient to avoid cross-excitation. In the Ramsey experiment, the laser beam was split by a 50:50 beam splitter. One arm of the beam was delayed by moving the retro-reflector (Thorlabs PS976/M) and then combined with the other undelayed beam using another 50:50 beam splitter.

### Diamond growth

The diamond sample was grown using a microwave plasma chemical vapour deposition technique. Detonation nanodiamonds (4–6) nm were dispersed on a silicon substrate and used as seeds for the diamond growth. The growth condition was 60 Torr, 950 W. The final size of the nanodiamonds was several hundreds of nanometres.

### Note added

In preparation of this manuscript, we noted a paper reporting a complementary method for measuring the Rabi oscillations^37^.

### Data availability

All summary data are included in the manuscript and Supplementary Information. Requests for more detailed data collected for this study are available from the corresponding author on request.

## Additional information

**How to cite this article:** Zhou, Y. *et al*. Coherent control of a strongly driven silicon vacancy optical transition in diamond. *Nat. Commun.* **8**, 14451 doi: 10.1038/ncomms14451 (2017).

**Publisher's note:** Springer Nature remains neutral with regard to jurisdictional claims in published maps and institutional affiliations.

## Supplementary Material

Supplementary Information Supplementary Figures, Supplementary Table, Supplementary Note and Supplementary References
