# Supplementary material for: Coherent control of a strongly driven silicon vacancy optical transition in diamond
Source: Nat Commun. 2017 Feb 20;8:14451. doi: 10.1038/ncomms14451 (PMC5321752; doi:10.1038/ncomms14451)
Supplement: Supplementary Information — Supplementary Figures, Supplementary Table, Supplementary Note and Supplementary References [file ncomms14451-s1.pdf]

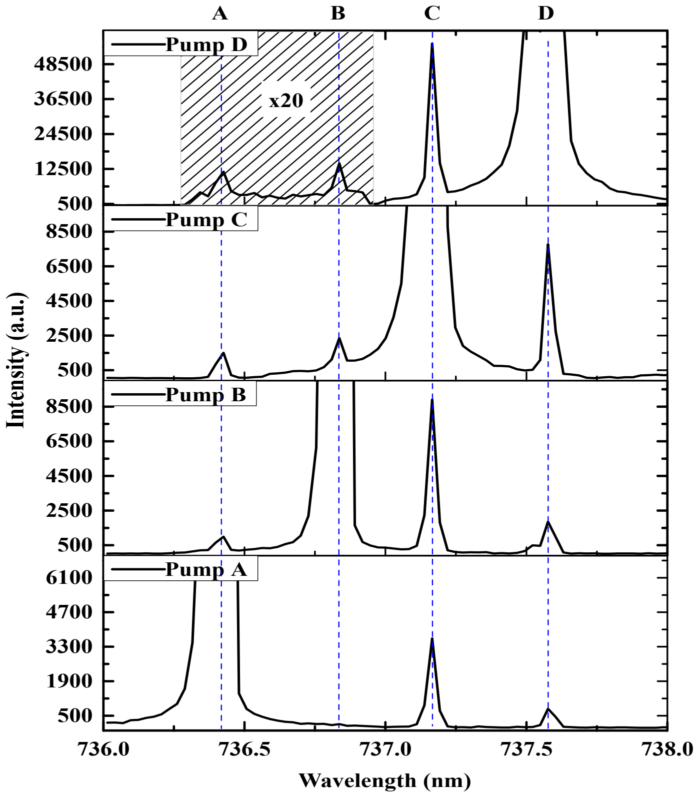

Supplementary Figure 1: **Fine structures of SiV in nano diamond.** In the resonant excitation scan map from 736 nm to 738 nm, there are tens of transitions belonging to different silicon vacancies. To identify four transitions belonging to one single silicon vacancy, we resonantly excite one transition using very low power (tens of nanowatts) and then collect the zero phonon lines of other thee transitions. To suppress residual counts from the excitation laser, we collect emission with polarization perpendicular to the input laser. In this figure, we show photoluminescence (PL) measurements of four resonantly excited transitions of a single silicon vacancy. Note that the spectrum intensity in the crosshatched area is twenty times its original value, to make the illustration clearer. As shown here, when excited with one transition, spectrum intensity at this wavelength is very strong due to the remaining excitation laser, however, more importantly, other three transitions are clear in the spectrum. We resonantly excite these four transitions respectively, and confirm that we have successfully identified the fine structures (A, B ,C, D) from a single silicon vacancy center. The spectrum intensity on the resonant wavelength is saturated due to the residual of the excitation laser. Three other transitions are visible and all wavelength match in the four figures. In the bottom picture, when excited with A transition, the peak at the B transition wavelength is very small, however, C and D are clear. C transition is used for the Mollow triplet experiment in the main text, since both of the ground state and excited state energy levels correspond to the lower one (with longer lifetime).

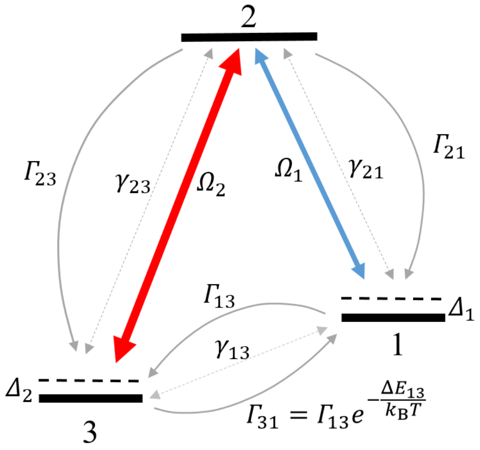

Supplementary Figure 2: **Energy level model of silicon vacancy (SiV) dynamics.** We model the dynamics of the silicon vacancy (SiV) using an open Λ system including two ground states labeled as state 1 and state 3 and one excited state labeled as state 2. The Ω_2 (Ω_1) is the laser Rabi frequency for transition C (D), Δ_2 (Δ_1) is the laser detuning for transition C (D), Γ_{ij} is the spontaneous emission (relaxation) rate between state j and state i, and γ_{ij} is the dephasing rate between state j and state i. The Λ system is driven between the ground states and the excited state. The transition C in the main text corresponds to optical transition between state 2 and state 3 while transition D corresponds to the optical transition between state 2 and state 1. The population of state 2 is proportional to the fluorescence intensity. The description of the Hamiltonian and the Lindblad master equation is given in Supplementary Note 1.

Supplementary Table 1: **Parameter values used in the theoretical model of SiV dynamics.** The values of the parameter used to obtain Figure 4e in the main text are shown in this table. The definition of each parameter is given in Supplementary Figure 2. The values of Ω_1, Γ_{13} and γ_{13} are chosen such that the measured normalized fluorescence intensity at $\Delta_2 = 0$ for various C transition power ranging from $\sim 2P_{\mathrm{sat}}$ to $\sim 40P_{\mathrm{sat}}$ fit the theoretical normalized intensity from the Lindbald master equation. The values of other parameters are chosen following the method described in Supplementary Note 1. These values are then used to get the simulated normalized fluorescence intensity for the whole experimental range of (Δ_1, Δ_2). As shown in Figure 4d&e in the main text, the result shows a good agreement between the simulated and measured values of fluorescence intensity.

Parameter name	Parameter value
Ω_1	2 GHz
Ω_2	2.7 GHz
$\Gamma_{21}(\Gamma_{23})$	270 MHz
$\gamma_{21}(\gamma_{23})$	350 MHz
Γ_{13}	280 MHz
γ_{13}	200 MHz

Supplementary Note 1. Lindblad master equation model

The Lindblad master equation can be written as

$$\frac{d\rho}{dt} = -\frac{i}{\hbar}[H_{\text{int}}, \rho] + \frac{1}{2}\sum[2C_{ij}\rho C_{ij}^{\dagger} - \{C_{ij}^{\dagger}C_{ij}, \rho\}] \tag{1}$$

where H_{int} is the Hamiltonian of the effective three-level system in the interaction picture after applying rotating wave approximation and C_{ij} is the collapse operator corresponding to transition between level i and j. The Lindblad master equation is solved for steady state solution by setting $\frac{d\rho}{dt} = 0$.

The Hamiltonian H_{int} of the three-level system that we considered here can be written as

$$H_{\text{int}} = \begin{bmatrix} 0 & \frac{\Omega_1}{2} & 0 \\ \frac{\Omega_1}{2} & \Delta_1 & \frac{\Omega_2}{2} \\ 0 & \frac{\Omega_2}{2} & \Delta_1 - \Delta_2 \end{bmatrix} \tag{2}$$

where Ω_2 (Ω_1) is the laser Rabi frequency for transition C (D) and Δ_2 (Δ_1) is the laser detuning for transition C (D).

The collapse operator C_{ij} can be expressed as

$$C_{ij} = \sqrt{\Gamma_{ij}}\,|j\rangle\langle i| \tag{3}$$

where $\Gamma_{ij}, i \neq j$ is the spontaneous emission (relaxation) rate between state j and state i, and Γ_{ii} is related to the dephasing rate γ_{ij} through relation $\gamma_{ij,i<j} = \frac{\Gamma_{ii}+\Gamma_{jj}}{2}$. In the case of three level system, Γ_{ii} can be expressed as

$$\Gamma_{11} = \gamma_{12} + \gamma_{13} - \gamma_{23} \tag{4}$$

$$\Gamma_{22} = \gamma_{12} - \gamma_{13} + \gamma_{23} \tag{5}$$

$$\Gamma_{33} = -\gamma_{12} + \gamma_{13} + \gamma_{23} \tag{6}$$

In order to reduce the number of free parameters in the model, the following is done:

- It is assumed that the relaxation rate and dephasing rate between level 2 and 1 are the same as those for level 2 and 3. That is $\Gamma_{21} = \Gamma_{23}$ and $\gamma_{21} = \gamma_{23}$. The values of $\Gamma_{21(23)}$ and $\gamma_{21(23)}$ then can be estimated from T_1 and T_2 of transition C using following relations

$$\Gamma_{21} = \Gamma_{23} = \frac{1}{2T_1} \tag{7}$$

$$\gamma_{21} = \gamma_{23} = \frac{1}{T_2} - \frac{1}{2T_1} \tag{8}$$

- Following the same argument used in [1], the thermalization assumption is used between state 1 and 3. Therefore $\mathit{\Gamma}_{31}$ can be written as $\mathit{\Gamma}_{31} = \mathit{\Gamma}_{13} e^{-\frac{\Delta E_{13}}{k_B T}}$ with ΔE_{13} is the energy difference between level 1 and 3 which is found to be equal to 951 μeV.

- The value of $\mathit{\Omega}_2$ is obtained experimentally (see Figure 3b in the main text).

Using this approach, there are only three fitting parameters: $\mathit{\Omega}_1$, $\mathit{\Gamma}_{13}$ and γ_{13}.

Supplementary References

1. Pingault, B., et al. All-optical formation of coherent dark states of silicon-vacancy spins in diamond. Phys. Rev. Lett. **113**, 263601 (2014)
